# First-line erlotinib in patients with advanced non-small-cell lung cancer unsuitable for chemotherapy (TOPICAL): a double-blind, placebo-controlled, phase 3 trial

**DOI:** 10.1016/S1470-2045(12)70412-6

**Published:** 2012-11

**Authors:** Siow Ming Lee, Iftekhar Khan, Sunil Upadhyay, Conrad Lewanski, Stephen Falk, Geraldine Skailes, Ernie Marshall, Penella J Woll, Matthew Hatton, Rohit Lal, Richard Jones, Elizabeth Toy, David Chao, Gary Middleton, Sue Bulley, Yenting Ngai, Robin Rudd, Allan Hackshaw, Chris Boshoff

**Affiliations:** a University College London Cancer Institute and University College London Hospitals, London, UK; b Cancer Research UK and University College London Cancer Trials Centre, University College London Cancer Institute, London, UK; c Scunthorpe General Hospital, Scunthorpe, UK; d Charing Cross Hospital, London, UK; e Yeovil District Hospital, Yeovil, UK; f Royal Lancaster Infirmary, Lancaster, UK; g Clatterbridge Centre for Oncology, Liverpool, UK; h University of Sheffield, Sheffield, UK; i Doncaster Royal Infirmary, Doncaster, UK; j Biomedical Research Centre, Guy's and St Thomas' Hospitals, London, UK; k Inverclyde Royal Hospital, Greenock, UK; l Royal Devon and Exeter Hospital, Exeter, UK; m North Middlesex Hospital, London, UK; n Royal Surrey County Hospital, Guildford, UK

## Abstract

**Background:**

Many patients with advanced non-small-cell lung cancer (NSCLC) receive only active supportive care because of poor performance status or presence of several comorbidities. We investigated whether erlotinib improves clinical outcome in these patients.

**Methods:**

TOPICAL was a double-blind, randomised, placebo-controlled, phase 3 trial, done at 78 centres in the UK. Eligibility criteria were newly diagnosed, pathologically confirmed NSCLC; stage IIIb or IV; chemotherapy naive; no symptomatic brain metastases; deemed unsuitable for chemotherapy because of poor (≥2) Eastern Cooperative Oncology Group performance status or presence of several comorbidities, or both; and estimated life expectancy of at least 8 weeks. Patients were randomly assigned (by phone call, in a 1:1 ratio, stratified by disease stage, performance status, smoking history, and centre, block size 10) to receive oral placebo or erlotinib (150 mg per day) until disease progression or unacceptable toxicity. Investigators, clinicians, and patients were masked to assignment. The primary endpoint was overall survival. Analyses were by intention to treat, and prespecified subgroup analyses included development of a rash due to erlotinib within 28 days of starting treatment. This study is registered, number ISRCTN 77383050.

**Findings:**

Between April 14, 2005, and April 1, 2009, we randomly assigned 350 patients to receive erlotinib and 320 to receive placebo. We followed up patients until March 31, 2011. 657 patients died; median overall survival did not differ between groups (erlotinib, 3·7 months, 95% CI 3·2–4·2, *vs* placebo, 3·6 months, 3·2–3·9; unadjusted hazard ratio [HR] 0·94, 95% CI 0·81–1·10, p=0·46). 59% (178 of 302) of patients assigned erlotinib and who were assessable at 1 month developed first-cycle rash, which was the only independent factor associated with overall survival. Patients with first-cycle rash had better overall survival (HR 0·76, 95% CI 0·63–0·92, p=0·0058), compared with placebo. Compared with placebo, overall survival seemed to be worse in the group that did not develop first-cycle rash (1·30, 1·05–1·61, p=0·017). Grade 3 or 4 diarrhoea was more common with erlotinib than placebo (8% [28 of 334] *vs* 1% [four of 313], p=0·0001), as was high-grade rash (23% [79 of 334] *vs* 2% [five of 313], p<0·0001); other adverse events were much the same between groups.

**Interpretation:**

Patients with NSCLC who are deemed unsuitable for chemotherapy could be given erlotinib. Patients who develop a first-cycle rash should continue to receive erlotinib, whereas those who do not have a rash after 28 days should discontinue erlotinib, because of the possibility of decreased survival.

**Funding:**

Cancer Research UK, Roche.

## Introduction

Lung cancer, the main cause of cancer-related death, accounts for nearly 1·4 million deaths worldwide every year, and has an annual incidence of more than 41 000 in the UK (age standardised incidence of 48 per 100 000).^1^ Mortality from lung cancer accounts for more than 452 000 deaths in China, 342 000 in Europe, and 162 000 in the USA.^1^ The number of lung-cancer deaths in developing countries is expected to increase during the next few decades such that by 2030 about 70% of tobacco-related deaths will occur in these countries. About 85–90% of patients with lung cancer have non-small-cell lung cancer (NSCLC), with most presenting with advanced or metastatic disease. Treatment guidelines recommend four to six cycles of first-line platinum-based doublet chemotherapy. However, most patients with advanced or metastatic NSCLC are elderly (median age 72 years^2^) and many receive only active supportive care, including palliative radiotherapy, because of physician choice, poor performance status, or presence of several comorbidities.^2^, ^3^ Therefore, despite the recommendation to treat these patients with platinum-based chemotherapy, only about 25% of elderly (age >65 years) US patients^3^ and 29% of newly diagnosed UK patients^2^ currently receive any cytotoxic treatment.

Erlotinib is an oral EGFR inhibitor that is associated with a significant survival benefit among patients with NSCLC when used as maintenance monotherapy after first-line chemotherapy or as second-line or third-line monotherapy.^4^, ^5^, ^6^ In chemotherapy-naive patients with activating *EGFR* mutations, erlotinib significantly improved progression-free survival compared with chemotherapy.^7^, ^8^

We did a large randomised trial to determine whether erlotinib monotherapy would be beneficial as first-line therapy in unselected patients with advanced NSCLC deemed unsuitable for chemotherapy.

## Methods

### Study design and participants

TOPICAL was a superiority phase 3, double-blind, randomised, placebo-controlled trial. Eligibility criteria were newly diagnosed, pathologically confirmed NSCLC; stage IIIb or IV disease; chemotherapy naive; no symptomatic brain metastases; deemed unsuitable for chemotherapy because of poor Eastern Cooperative Oncology Group (ECOG) performance status (PS ≥2) or presence of several comorbidities (including impaired renal function with creatinine clearance <60 mL/min), or both; and estimated life expectancy of at least 8 weeks. Such patients do not normally receive chemotherapy. Other inclusion criteria were age older than 18 years, diagnosis within the past 62 days, able to take oral medication, and using effective contraception if appropriate. Exclusion criteria were previous treatment with any biological anticancer therapy; previous palliative radiotherapy (except to bone metastases, within the previous 2 weeks); pregnant or lactating women; evidence of significant laboratory finding or concurrent uncontrolled medical illness judged to potentially interfere with the trial treatment; and present treatment with a COX-2 inhibitor. We obtained multicentre and local research ethics approvals. All patients provided written informed consent.

### Randomisation and masking

Randomisation was done by site staff telephoning the Cancer Research UK and University College London Cancer Trials Centre. Patients were randomised in a 1:1 ratio to either once daily oral erlotinib (150 mg tablets) or matching placebo, with a computer generated sequence, stratified by disease stage (IIIb, IV), performance status (0–1, 2, 3), smoking history (never, current/former), and centre (78 sites), with a block size of 10. All investigators, clinicians, and patients were masked to assignment.

### Procedures

Patients were to take oral erlotinib or matching placebo daily, 1 h or more before food, or 2 h after food. The dose could be reduced to 100 mg, then 50 mg in cases of substantial toxic effects. Treatment continued until disease progression, adverse side-effects judged by the treating clinician to warrant discontinuation, or patient withdrawal. Patients continued to receive active supportive care, including palliative radiotherapy, at the discretion of their clinician.

The primary endpoint was overall survival, measured from the date of randomisation until death from any cause. Secondary endpoints were progression-free survival (time until progression or death from any cause), tumour response (according to Response Evaluation Criteria in Solid Tumours), adverse events, and quality of life. Patients who survived or who did not have progression were censored at the date they were last known to be alive.

Within 4 weeks before randomisation patients had a physical examination, assessment of comorbidities with the Charlson comorbidity index (CCI), blood count and biochemistry, chest radiograph, and CT of the chest and abdomen. Clinicians did bone and brain scans when clinically indicated. Patients completed quality-of-life assessments (European Organisation for Research and Treatment of Cancer QLQ C-30 and LC14, and EuroQol 5-dimensional scale) at baseline, monthly during the first year, then 18 and 24 months after randomisation. Presence of several comorbidities was defined as CCI of 3 or more.

Clinicians did physical examinations, including assessment of performance status, development of rash, and adverse events (with National Cancer Institute of Canada Common Toxicity Criteria for Adverse Events, version 3.0), and a chest radiograph every month for the first 12 months, and every 2 months thereafter. We graded rash with the criteria: erythema alone (A), erythema with papules (B), erythema with papules and pustules (C), and erythema with papules and confluent pustules (D). CT scans were done at 3 and 6 months and when clinically indicated—eg, after abnormal chest radiographs.

For translational research, we collected blood samples and diagnostic biopsy material (paraffin blocks) before starting treatment. Purification and assessment of quality and quantity of tumour DNA were done with Wizard genomic DNA purification kit (Promega, Madison, WI, USA). We used the Sequenom OncoCarta Panel v1.0 service (Sequenom, Germany) to do a sensitive (ie, detection of low abundance mutations corresponding to mutation frequencies of up to 10%) analysis of 238 known cancer mutations in 19 genes, including activating *EGFR* mutations and *KRAS* mutations.

### Statistical analysis

The target sample size was 664 patients, on the basis of the primary study objective to detect an increase in 1 year overall survival from 10% with placebo to 17·5% with erlotinib (equivalent to a HR of 0·75 and much the same as that achieved with chemotherapy *vs* supportive care^9^), with 90% power and 5% two-sided test of significance.

We did analyses (with SAS version 9.2) by intention to treat, with Kaplan-Meier curves and Cox proportional hazards regression (hazard ratios [HRs] estimated with maximum likelihood methods), unless otherwise specified (the proportional hazards assumption was met for overall survival and progression-free survival). We used the maximum grade for each type of adverse event for each patient. We analysed quality of life with mixed effects models for repeated measures with baseline values as a covariate. We did model adequacy checks for continuous variables, including those for departures from normality assumptions, with residual plots and normal probability plots. We calculated compliance to study treatment by dividing the total number of tablets taken (equivalent to number of days on study drug) by the number of days from randomisation to death, progression, or when treatment was stopped early, and expressed results as a percentage. Preplanned subgroup analyses included sex, histological examination, activating *EGFR* or *KRAS* mutation, stage, smoking status, ECOG score, and development of first-cycle rash.

This study is registered, number ISRCTN 77383050.

### Role of the funding source

The trial was funded by Cancer Research UK (C1438/A4147), with an educational grant from Roche for the translational studies, but neither were involved in trial design, data analyses or interpretation, or writing of this report. Additional support came from the University College London and University College London Hospital Biomedical Research Centre, who had no role in study design, data collection, analysis, or interpretation, or writing of the report. The trial sponsor was University College London, who were involved trial design, data collection, analyses, and interpretation, and writing of the report. SML, IK, and AH had access to the full raw data. The corresponding author had the final responsibility for the decision to submit for publication.

## Results

**Figure 1:**
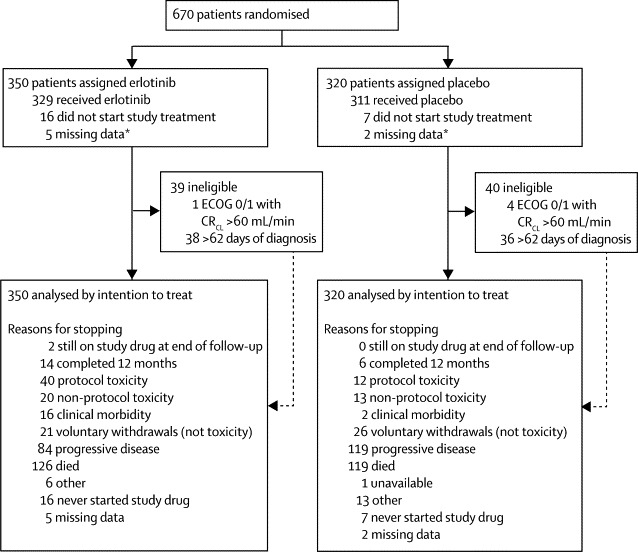
Trial profile *Patients with no recorded start date of study drug or dosing details. ECOG=Eastern Cooperative Oncology Group performance status. CR_CL_=creatinine clearance.

We recruited 670 patients from 78 centres from the UK National Cancer Research Network between April 14, 2005, and April 1, 2009. Our randomisation procedure gave 936 cells and by chance the first allocation in each cell for several centres was erlotinib, producing an imbalance in the number randomised to each group; 350 participants were assigned to receive erlotinib and 320 placebo (figure 1). We followed up patients until March 31, 2011. Baseline characteristics were balanced, including the comorbidity index (table 1).

**Table 1:** Baseline characteristics

		**Erlotinib (N=350)**	**Placebo (N=320)**
Age at randomisation			
	Median (years)	77 (72–82)	77 (72–81)
	≥75 years	220 (63%)	203 (63%)
Sex			
	Men	215 (61%)	194 (61%)
	Women	135 (39%)	126 (39%)
ECOG performance status			
	0–1	54 (15%)*	52 (16%)*
	2	194 (55%)	178 (56%)
	3	102 (29%)	90 (28%)
Stage			
	IIIb	127 (36%)	107 (33%)
	IV	223 (64%)	213 (67%)
Cell type			
	Adenocarcinoma	133 (38%)	123 (38%)
	Squamous	136 (39%)	127 (40%)
	Large cell	15 (4%)	15 (5%)
	Other	66 (19%)	55 (17%)
Ethnic origin			
	White	336 (96%)	314 (98%)
	Asian	7 (2%)	3 (1%)
	Other	7 (2%)	3 (1%)
Smoking status			
	Smoker	124 (35%)	119 (37%)
	Ex-smoker	207 (59%)	183 (57%)
	Never smoked	19 (5%)	18 (6%)
	Median pack-years (current or ex-smoker)†	40 (24–60)	38 (21–55)
Total CCI‡			
	Median	4·0 (3–5)	4·0 (3–5)
	0	3 (1%)	1 (<1%)
	1	4 (1%)	6 (2%)
	2	17 (5%)	18 (6%)
	3	72 (21%)	70 (22%)
	≥4	241 (69%)	219 (68%)
	Unknown	13 (4%)	6 (2%)

Data are n (%) or median (IQR). ECOG=Eastern Cooperative Oncology Group. CCI=Charlson comorbidity index.

* All patients with a performance score of 0–1 had comorbidities, ie, 92% (98 of 106) had CCI scores of ≥3, 95% (101 of 106) had creatinine clearance <60 mL/min, and a median age of 81 years with 81% (86 of 106) aged >75 years old. Characteristics were well balanced between groups.

† One pack-year is defined as 20 cigarettes (one pack) smoked per day for one year.

‡ The CCI is measured on a 0 to 37 scale: 0 means no comorbidities whereas a high score suggests patients have more severe comorbidities. Patients with a score ≥4 are deemed to have serious comorbidity.

16 patients assigned erlotinib and seven patients assigned placebo did not start study treatment, because patients had died or progressed beforehand. Compliance to study treatment (defined as patients who took their tablets for ≥75% of the time that they were in the trial, until they died or stopped treatment early), was 58% (204 of 350) for erlotinib and 63% (203 of 320) for placebo (median compliance was 88% [IQR 0–98] for erlotinib and 86% [0–96] for placebo, appendix p 4). Figure 1 shows that the main reasons for stopping trial treatment were toxic effects or disease progression. After discontinuation of study treatment, the most common subsequent therapy was palliative radiotherapy, which was given to 34% (119 of 350) of patients assigned erlotinib and 36% (114 of 320) of those assigned placebo. Only 2% (12 of 670) of patients received a tyrosine-kinase inhibitor (n=3) or chemotherapy (n=9) after disease progression, seven in the placebo group and five in the erlotinib group. The only tyrosine-kinase inhibitor used was erlotinib.

**Figure 2:**
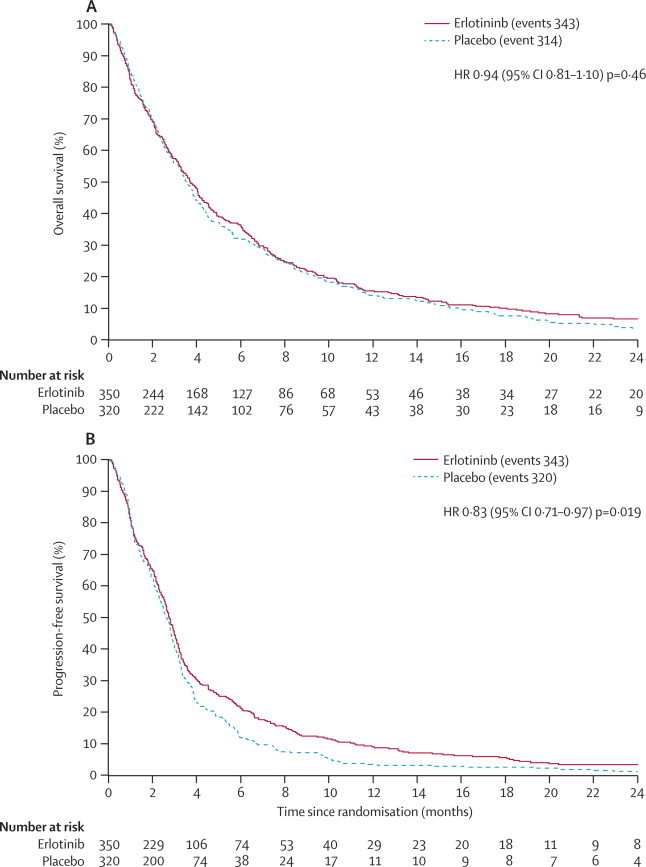
Overall survival and progression-free survival for all patients HR=hazard ratio.

657 patients died; 314 in the placebo group and 343 in the erlotinib group, with 93% (608 of 657) of deaths attributed to progressive disease. Figure 2 shows Kaplan-Meier curves for overall survival and progression-free survival. We identified no difference in overall survival among all patients; median overall survival was 3·7 months (95% CI 3·2–4·2) in the erlotinib group versus 3·6 months (3·2–3·9) in the placebo group (unadjusted HR 0·94, 95% CI 0·81–1·10, p=0·46; adjusted HR 0·92, 0·78–1·07, p=0·31). 1 year overall survival was 14% (95% CI 10–18) with placebo versus 15% (12–19) with erlotinib.

We identified a significant improvement in progression-free survival with erlotinib (unadjusted HR 0·83, 95% CI 0·71–0·97, p=0·019; adjusted HR 0·80, 0·68–0·93, p=0·0054); median progression-free survival was 2·8 months (95% CI 2·6–3·0) with erlotinib versus 2·6 months (2·4–2·9) with placebo (figure 2B). Tumour response rates are shown in the appendix (p 5); 4% (15 of 350) of patients in the erlotinib group and 2% (seven of 320) of those in the placebo group had a complete or partial tumour response.

Among the 647 patients who started study treatment, the occurrence of any grade 3–4 adverse event was 75% (252 of 334) with erlotinib and 70% (219 of 313) with placebo (p=0·12, table 2). More patients assigned erlotinib had rash at any time and of any grade than did those assigned placebo (56% [188 of 334] *vs* 15% [46 of 313] p<0·0001). 24% (79 of 334) of patients assigned erlotinib had a high-grade (C or D) rash versus 2% (five of 313) of those assigned to placebo (p<0·0001). Significantly more patients assigned erlotinib had diarrhoea of grade 3–4 than did those assigned placebo (table 2). 21% (69 of 334) of patients assigned erlotinib had diarrhoea of grade 1–2 versus 8% (24 of 313) for placebo (p<0·0001), and rash and diarrhoea mainly occurred within the first month of treatment. Other adverse events were much the same between the groups.

**Table 2:** Adverse events among all patients who started study treatment

	**Erlotinib (N=334)**	**Placebo (N=313)**	**p value**
**Any event (maximum grade**			
1	20 (6%)	26 (8%)	..
2	28 (8%)	32 (10%)	..
3	132 (40%)	99 (32%)	..
4	120 (36%)	120 (38%)	..
Any (grade 1–4)	300 (90%)	277 (88%)	0·18
Any (grade 3–4)*	252 (75%)	219 (70%)	0·12
Any (grade 3–4) excluding rash and diarrhoea	145 (43%)	210 (67%)	0·11
**Rash (maximum grade)**			
No rash or grade 0	67 (20%)	201 (64%)	..
A (erythema alone)	50 (15%)	33 (11%)	0·09
B (erythema with papules)	59 (18%)	8 (3%)	<0·0001
C (erythema with papules and pustules)	65 (19%)	5 (2%)	<0·0001
D (erythema with papules and confluent pustules)	14 (4%)	0	<0·0001
Data unavailable because of death†	36 (11%)	31 (10%)	..
Missing data‡	43 (13%)	35 (11%)	..
**Dyspnoea**			
Grade 3 (dyspnoea on walking ≥100 yards)	91 (27%)	87 (28%)	..
Grade 4 (dyspnoea on mild exertion)	105 (31%)	112 (36%)	..
Grade 3–4	196 (59%)	199 (64%)	0·18
**Specific adverse events (grade 3 or 4 only)**			
Fatigue	77 (23%)	73 (23%)	..
Diarrhoea	28 (8%)	4 (1%)	<0·0001
Anorexia	18 (5%)	15 (5%)	..
Anaemia	6 (2%)	3 (1%)	..
Nausea	5 (1%)	6 (2%)	..
Pneumonitis	5 (1%)	1 (<1%)	..
Rigor chills	4 (1%)	0	..
Stomatitis	4 (1%)	0	..
Ocular	3 (1%)	0	..
Vomiting	2 (1%)	1 (<1%)	..
Constipation	1 (<1%)	5 (2%)	0·08
Headache	0	2 (1%)	..

Data are n (%).

* Results when all 670 patients were used were 72% for elotinib *vs* 69% for placebo (p=0·43).

† For patients who died before the first month assessment, rash could not be recorded.

‡ No rash data available at any time, but patient was alive for >1 month.

Patients assigned erlotinib had significantly improved quality of life for two of five functional scales (cognitive [p=0·0072] and physical functioning [p=0·0024]) and for six of 18 symptoms (pain [p=0·0018], dyspnoea [p<0·0001], chest pain [p<0·0001], hoarseness [p<0·0001], constipation [p<0·0001], and financial problems [p<0·0001]); appendix p 6). We recorded increased diarrhoea (mean difference 15·1), hair loss (12·6), sore mouth (6·4), and decreased constipation (–9·4) more frequently in patients assigned erlotinib than in those assigned placebo.

**Figure 3:**
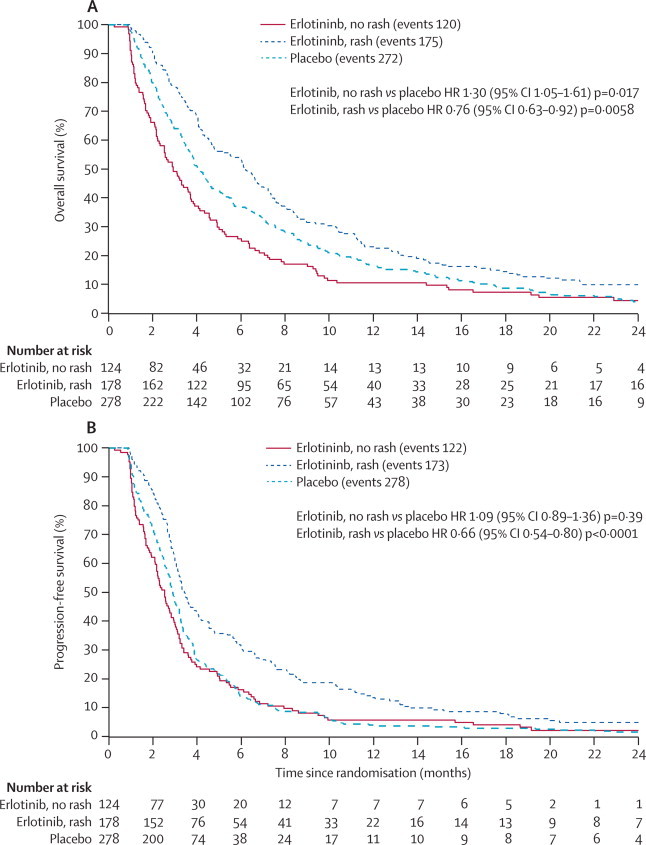
Overall survival and progression-free survival according to whether patients on erlotinib developed first-cycle rash or not HR=hazard ratio.

In prespecified subgroup analyses, first-cycle rash among patients assigned erlotinib was the only significant independent factor (HR 0·24, 95% CI 0·16–0·35, p<0·0001; table 3) associated with overall survival, from a stepwise selection multivariate analysis containing rash, sex, histological examination, ECOG score, stage, and smoking status. We identified a clear association between progression-free survival and overall survival and whether patients assigned erlotinib developed rash or not (figure 3). Patients were classified as having first-cycle rash (any grade) when the rash occurred within the first 28 days, which was when we made the first assessment of rash. Of the 647 patients who started study treatment, 67 who died before this assessment were excluded from this subgroup analysis, to avoid bias by classification of them as not having rash. 59% (178 of 302) of patients assigned erlotinib developed first-cycle rash (compared with 5% [15 of 278] assigned placebo), and we recorded a positive correlation between overall survival (p<0·0001) and progression-free survival (p<0·0001) with increasing rash severity.

**Table 3:** Summary of multivariate analysis for overall survival and progression-free survival

	**Hazard ratio (95% CI)**	**p value**
Predictor variables for overall survival*		
Rash	0·24 (0·16–0·35)‡	<0·0001
Women *vs* men	0·81 (0·59–1·01)	0·17
Adenocarcinoma *vs* non-adenocarcinoma	0·92 (0·66–1·29)	0·64
ECOG 0–1 *vs* 2–3	0·87 (0·53–1·40)	0·55
Stage IIIb *vs* IV	0·84 (0·61–1·10)	0·23
Ex-smoker *vs* smoker	0·92 (0·71–1·18)	0·51
Never smoker *vs* smoker	0·64 (0·36–1·14)	0·13
**Predictor variables for progression-free survival**†		
Rash	0·41 (0·27–0·60)‡	<0·0001
Women *vs* men	0·74 (0·44–1·03)	0·058
Adenocarcinoma *vs* non-adenocarcinoma	0·99 (0·71–1·37)	0·95
ECOG 0–1 *vs* 2–3	0·91 (0·56–1·48)	0·70
Stage IIIb *vs* IV	0·83 (0·59–1·09)	0·21
Ex-smoker *vs* smoker	0·98 (0·76–1·27)	0·88
Never smoker *vs* smoker	0·62 (0·35–1·10)	0·10

* For overall survival, p values from overall tests were p=0·17 for sex, p=0·62 for ECOG, p=0·84 for histological examination, p=0·23 for stage, and p=0·35 for smoking status.

† For progression-free survival, p values from overall tests were p=0·06 for sex, p=0·86 for ECOG, p=0·79 for histological examination, p=0·21 for stage, and p=0·28 for smoking status.

‡ Result after a stepwise selection; rash remained the only significant variable in the model for overall survival and progression-free survival.

Among patients assigned erlotinib who developed rash (compared with all those assigned placebo), overall survival was significantly longer (HR 0·76, 95% CI 0·63–0·92, p=0·0058), as was progression-free survival (0·66, 0·54–0·80, p<0·0001). Hazard ratios for patients assigned erlotinib who did not develop rash, compared with placebo, were 1·30 (95% CI 1·05–1·61, p=0·017) for overall survival, and 1·09 (0·89–1·36, p=0·39) for progression-free survival, neither of which overlapped the corresponding intervals for patients who were treated with erlotinib and developed rash, indicating that they were significantly different (ie, evidence for an interaction). Median overall survival was 6·2 months (95% CI 4·8–7·2) for the erlotinib and rash group, 2·9 months (2·3–3·7) for the erlotinib without rash group, and 4·1 months (3·7–4·6) for placebo. 1 year overall survival was 24% (95% CI 17–29) for erlotinib and rash, 10% (5–16) for erlotinib without rash, and 18% (12–21) for placebo. For erlotinib and rash compared with placebo, the number needed to treat to save one life at 6 months was seven and at 12 months was 17. Median progression-free survival was 3·4 months (95% CI 3·1–4·0) for erlotinib and rash, 2·5 months (2·2–2·8) for erlotinib without rash, and 2·9 months (2·7–3·2) for placebo.

We identified overall survival benefits in patients who developed first-cycle rash in all subgroups including those with the worst characteristics: ECOG performance status 3, overall survival HR 0·58 (95% CI 0·38–0·89, p=0·012) and progression-free survival HR 0·41 (0·26–0·65, p<0·0001); stage IV, overall survival HR 0·66 (0·52–0·84, p<0·0001) and progression-free survival HR 0·56 (0·44–0·72, p=0·0009); and age 75 years or older, overall survival HR 0·77 (0·61–0·97, p=0·028) and progression-free survival HR 0·71 (0·56–0·89, p=0·0032). Kaplan-Meier curves for overall survival were much the same for patients assigned placebo who did or did not develop rash (p=0·35, data not shown).

Patients assigned erlotinib who developed first-cycle rash had a higher occurrence of fatigue and diarrhoea than did those assigned erlotinib who did not develop rash (appendix p 7). The proportions with grade 3–4 fatigue were 30% (53 of 178) for erlotinib and rash, 19% (24 of 124) for erlotinib without rash, and 26% (72 of 278) for placebo. For grade 3–4 diarrhoea, the proportions were 11% (20 of 178) for erlotinib and rash, 6% (eight of 124) for erlotinib without rash, and 1% (four of 278) for placebo. When we excluded first-cycle rash, the proportion of patients reporting at least four grade 3–4 adverse events was 47% (84 of 178) for erlotinib and rash, 19% (23 of 124) for erlotinib without rash, and 36% (99 of 278) for placebo (appendix p 8). Among patients assigned erlotinib who developed rash, we recorded much the same effects on quality of life as we did in the main analysis, with dyspnoea (–9·3) and chest pain (–6·7) significantly improved compared with patients without rash (appendix p 9).

Appendix p 2 shows the results of the other prespecified subgroup analyses, according to the presence or absence of rash in participants assigned erlotinib. Among patients without rash, we identified no evidence of benefit, and overall survival might be worse for some subgroups, such as men (HR 1·52, 95% CI 1·13–2·04, p=0·0046), or ECOG performance status 3 (1·69, 1·11–2·58, p=0·014). Patients assigned erlotinib who developed rash had longer overall survival and progression-free survival than did those assigned erlotinib who did not develop rash irrespective of baseline characteristics, although some subgroups, including women (overall survival HR 0·66, 95% CI 0·48–0·90, p=0·0090) and patients with adenocarcinoma (0·55, 0·39–0·77, p=0·00049) seemed to have a greater benefit than did other subgroups. Median overall survival for erlotinib and rash versus placebo was 8·1 months (95% CI 6·2–10·4) versus 4·5 months (3·9–5·6) for women, and 4·9 months (4·1–6·3) versus 3·8 months (3·3–4·4) for men. However, the interaction test between sex and treatment was not significant (p=0·29), and some of this difference might be explained by a higher compliance to erlotinib in women (68%, 81 of 119) than in men (52%, 101 of 195). Appendix p 10 shows further results for overall survival and progression-free survival according to sex and histological examination.

In our biological substudy, DNA was available for 390 patients out of 398 (58% of total study population) who provided material. Occurrence of the activating *EGFR* mutation in the study population was only 7% (28 of 390), and 19% (73 of 390) for *KRAS*. Of the 28 *EGFR* mutation-positive samples, 11 had exon 19 deletions, ten had a mutation at exon 21 (858Leu→Arg), and seven had other mutations. In these patients, median overall survival was 10·4 months (95% CI 5·5–15·1) for erlotinib (n=17) versus 3·7 months (0·3–49·3) for placebo (n=11). Median progression-free survival was 4·8 months (1·6–8·8) for erlotinib and 2·9 months (0·3–10·1) for placebo. All patients with an *EGFR* mutation who were assigned erlotinib developed rash. Among patients with wild-type *EGFR* who were assigned erlotinib and developed rash (n=94), HR for overall survival was 0·86 (95% CI 0·66–1·12, p=0·27) and for progression-free survival was 0·69 (0·53–0·90, p=0·0070; appendix p 2). HR for those assigned erlotinib who did not develop rash (n=67) was 1·28 (0·95–1·72, p=0·10) for overall survival and 1·05 (0·78–1·41, p=0·74) for progression-free survival.

*KRAS* mutation-positivity was not associated with overall survival or progression-free survival. Among those who were *KRAS* mutation-positive, median overall survival was 4·2 months (95% CI 1·8–6·2) for erlotinib (n=35) versus 3·6 months (1·9–4·4) for placebo (n=38); median progression-free survival was 3·5 months (1·7–4·8) versus 2·7 months (1·8–3·9). Patients with wild-type *KRAS* had median overall survival of 3·7 months (2·8–4·2) for erlotinib (n=210) versus 3·4 months (2·7–4·3) for placebo (n=180); median progression-free survival was 2·7 months (2·2–2–9) for erlotinib and 2·6 months (2·3–2·9) for placebo. The presence or absence of first-cycle rash did not affect the results (data not shown).

## Discussion

First-line treatment with erlotinib did not improve overall survival in all unselected patients with advanced NSCLC deemed unsuitable for chemotherapy treatment, who usually have a poor prognosis (about 3–4 months median overall survival^10^). However, erlotinib did improve progression-free survival. Additionally, in prespecified subgroup analyses, compared with placebo, erlotinib significantly improved both overall survival and progression-free survival for patients who developed a first-cycle rash; median overall survival in this group increased by 2·1 months, from 4·1 months to 6·2 months. Multivariate analysis among patients assigned erlotinib confirmed that rash was the only significant predictor of overall survival. First-cycle rash has been studied elsewhere,^11^ and in TOPICAL 95% of patients assigned erlotinib who developed rash did so within 28 days (much the same as 90% within 25 days in the BR.21 study^12^). Rash itself might be a predictive factor, irrespective of treatment. However, few patients assigned placebo developed first-cycle rash (5% compared with 59% assigned erlotinib), and we identified no difference in survival in patients assigned placebo who had rash compared with those who did not. Few patients were lost to follow-up because survival is short and patients with lung cancer in the UK are seen regularly and remain under the care of a hospital physician.

A limitation of our study was that the incidence of *EGFR* mutation was only 7% and a quarter of them were uncommon mutations. Most *EGFR* mutation studies focus on so-called hot spot analysis, looking only for common alterations, short deletions in exon 19, or the 858Leu→Arg point mutation in exon 21. Our population were mostly elderly, white, present or ex-smokers, and such groups might have a different mutation spectrum profile, which further studies could examine. Nevertheless, although analyses were based on only 28 patients, overall survival and progression-free survival were improved in patients with an *EGFR* mutation who were assigned erlotinib, consistent with gefitinib in patients with poor performance status.^13^ However, patients with tumours showing wild-type *EGFR* also showed benefit when they developed an erlotinib-related rash. The BR.21^6^ (second and third line treatment) and SATURN^5^ trials (maintenance erlotinib after first-line chemotherapy), similarly reported that erlotinib was effective in predominantly good performance patients with wild-type *EGFR* (though BR.21 included some patients with performance status 2–3). We planned TOPICAL in 2002, and did not mandate compulsory tissue collection. In the IPASS study,^14^ only 56% (683) of patients provided samples with *EGFR* mutation data available in 36% (437) of the 1217 patients randomised. This proportion was much the same as in our trial where samples from 58% of patients had sufficient DNA for analysis.

Another limitation of our study is that we did routine CT scans at 3 and 6 months, whereas some other studies have used CT scans every 6–8 weeks, so our schedule might be deemed suboptimum for assessment of progression-free survival. However, the monthly chest radiographs would trigger a CT scan before the protocol timelines, and overall survival was the primary trial endpoint. Also, we included patients of performance status 2, for whom, on the basis of evidence reported in 2005,^15^ chemotherapy could improve survival, and a phase 2 study^16^ has shown that doublet chemotherapy has a better survival than single agent chemotherapy. However, when TOPICAL was designed, provision of chemotherapy to such patients was not routine practice because many studies with second and third generation chemotherapy had not shown survival benefit, and many of these patients had serious adverse events.^17^, ^18^ A limitation of many of the more recent chemotherapy trials is that median age of patients of performance status 2 is 65 years or younger, median survival is much the same as for patients of performance status 0–1, and many go on to receive second-line treatment, suggesting that they are a highly selected group. By contrast, in TOPICAL median age was 77 years, 90% of participants had several comorbidities, and less than 2% were given second-line treatment, which is indicative of the real-world scenario that lung cancer is a disease of elderly people with comorbidities, and many of these patients still continue to be treated with best supportive care worldwide, including palliative radiotherapy.^19^

When TOPICAL was designed some evidence already suggested that development of a rash during erlotinib therapy could be associated with improved outcomes across several cancers including NSCLC.^20^ With this knowledge, we developed a scoring system for rash as part of the TOPICAL protocol, and specified a preplanned subgroup analysis. Our phase 3 results confirm these early findings. Furthermore, retrospective analyses of two phase 3 NSCLC trials show a positive correlation between rash and treatment effect on survival: the BR.21 trial^12^ of erlotinib and the FLEX trial^11^ of cetuximab, an anti-EGFR antibody, when added to first-line chemotherapy. Researchers have recorded much the same findings in trials of pancreatic cancer, locally advanced head and neck cancer, and metastatic colorectal cancer.^12^, ^21^, ^22^ Currently no clear biological explanations link erlotinib activity with rash, but rash probably represents greater uptake of the drug. Smokers have a lower plasma concentration of erlotinib than do non-smokers.^23^, ^24^ Smokers also have a lower incidence of skin rash and have less clinical benefit from EGFR inhibitors compared with non-smokers.^23^, ^24^ This finding was confirmed in TOPICAL because present smokers in the erlotinib group were less likely to develop rash when compared with former smokers (odds ratio 0·29, 95% CI 0·18–0·48) or never-smokers (0·20, 0·06–0·66). Increased occurrence of skin rash has been identified in a phase 2 dose escalation study of erlotinib, although another study suggested that dose-escalation does not improve incidence or outcome.^25^, ^26^ Alternatively, skin rash might be a surrogate marker of patients able to mount antitumour immune response. Data increasingly suggest the importance of the host immune system in control of cancer cell growth after tyrosine-kinase inhibitor treatments.^27^, ^28^ As reported with other tyrosine-kinase inhibitors, erlotinib could enhance cytotoxic T-cell infiltration.

Drug uptake and cytotoxic T-cell infiltration could be linked, with increased uptake of drug causing increased EGFR blockade and cell killing, resulting in improved host immune response. Future studies could examine whether combining erlotinib and immunomodulatory agents can further fuel a more robust immunological response, increase the severity and incidence of skin rash, and potentially further improve durability of response, progression-free survival, and overall survival in this group of patients who are deemed not suitable for chemotherapy.

A phase 2 study^10^ of gefitinib (n=201) compared with placebo (INSTEP) in a group of patients with similar characteristics to those in TOPICAL showed no statistical difference in survival with a HR of 0·84 (95% CI 0·62–1·15), but we identified a suggestion of a benefit among patients with high EGFR gene copy number determined by fluorescence in-situ hybridisation (HR 0·44, 95% CI 0·17–1·12). 34 of the 100 patients in the gefitinib group developed rash, and none with placebo, but the investigators did not analyse this finding. The dose of gefitinib might have been subtherapeutic in patients with wild-type *EGFR* tumours, and this notion is supported by the lower than expected proportion who developed rash. Another phase 2 trial^29^ of only patients of performance status 2 (n=103) with a median age of less than 70 years compared erlotinib with carboplatin and paclitaxel, and showed that progression-free survival was better with chemotherapy than with erlotinib but progression-free survival did not differ between patients assigned erlotinib who developed rash compared with those who were assigned chemotherapy (HR 0·94, 95% CI 0·56–1·59). In TOPICAL, erlotinib seemed to have a greater effect in some subgroups than in others (eg, median overall survival was improved by 3·6 months for women with rash and 1·1 months for men with rash). Previous studies have also reported sex–treatment interactions with tyrosine-kinase inhibitors and other chemotherapies, where female patients benefited more than men.^30^

Diarrhoea, hair loss, and fatigue were expected adverse events associated with erlotinib, and their severity was usually mild to moderate. Overall, occurrence and severity of adverse events in TOPICAL were much the same as those in the SATURN and BR.21 trials despite our population being predominantly elderly patients with poor performance status (ECOG 2–3).^5^, ^6^ Taking erlotinib tablets at home should be more convenient to patients compared with treatments that require administration in hospital. Together with radiological assessment, first cycle erlotinib-induced rash could be used to select patients who are likely to benefit from continuous treatment. We believe that patients with poor performance status and activating *EGFR* mutation tumours should receive erlotinib or gefitinib, in line with the established evidence in patients with good performance status, and supported by the finding that the small number of these patients in TOPICAL all developed rash.^7^, ^8^, ^14^

Our data suggest patients with *EGFR* wild-type or unknown *EGFR* status tumours could start erlotinib but they should discontinue if they do not develop rash within 28 days, because these patients had no benefit in overall survival or progression-free survival, and in some subgroups overall survival could be reduced if erlotinib were taken continuously (panel). The reasons for this finding are unknown but for some of our patients (especially men and those with ECOG performance status of 3), the disease might be so advanced that any toxic treatment could accelerate deaths. Researchers have recorded deleterious effects of anti-EGFR in some patients treated with erlotinib or cetuximab.^31^, ^32^ A strategy of use of first-cycle erlotinib-induced rash to select patients with poor performance status for continued treatment could substantially improve cost effectiveness. Erlotinib is expensive: a 30-day pack costs about £1650 in the UK ($4740 in the USA). Future analyses of TOPICAL will examine the cost effectiveness of various approaches, and compare quality adjusted life-years. Second and third line erlotinib is marginally cost-effective compared with best supportive care, therefore first-line erlotinib could be more cost-effective.^33^

Panel **Research in context**

**Systematic review**

We did not do a systematic review of scientific literature before designing the TOPICAL trial (in 2002), because at the time there were no other studies of first-line EGFR inhibitors in patients with advanced non-small-cell lung cancer (NSCLC) who were elderly or had poor performance status. These patients are often excluded from first-line chemotherapy trials.

Lung cancer is predominantly a disease of elderly people, and elderly patients with advanced NSCLC deemed unsuitable for chemotherapy with poor performance status (2 and 3) or several comorbidities, or both, remain a major challenge. Since TOPICAL, no phase 3 trials of first-line monotherapy for this group have been reported.

**Interpretation**

The findings of TOPICAL and other trials^5^, ^6^, ^7^, ^8^ show that erlotinib is an important treatment for patients with NSCLC in various clinical settings, including as maintenance after first-line chemotherapy, as second-line or third-line monotherapy in unselected patients with advanced NSCLC, and in chemotherapy-naive patients with activating *EGFR* mutations. However, unlike the TOPICAL patients, most of the patients in other studies had good performance status.

The clinical implications of the TOPICAL trial are that patients who are deemed unsuitable for chemotherapy could be given erlotinib. Those who have *EGFR* mutation-positive tumours could receive continuous erlotinib; whereas those who have wild-type tumours should discontinue erlotinib after about 28 days if they do not develop a rash because of the possibility of decreased survival.

*EGFR* testing has now become standard of care to select patients who are *EGFR* mutation-positive for treatment with tyrosine-kinase inhibitors. If erlotinib is to become a standard therapy for patients who have *EGFR* wild-type tumours and are unsuitable for chemotherapy (93% in our trial) it should be in a selected population. Prospective studies are needed to increase our understanding of the biological mechanism linking rash and erlotinib benefit, including dose-escalation studies or studies of the relation between rash and tumour *EGFR* copy number.^34^ Further translational research with our biological samples might identify candidate markers for erlotinib-induced rash that can preselect patients for treatment with a marker measured at baseline, without having to treat all patients for 4 weeks, and then discontinue those without rash.
