# Supplementary material for: First-line erlotinib in patients with advanced non-small-cell lung cancer unsuitable for chemotherapy (TOPICAL): a double-blind, placebo-controlled, phase 3 trial
Source: Lancet Oncol. 2012 Nov;13(11):1161–70. doi: 10.1016/S1470-2045(12)70412-6 (PMC3488187; doi:10.1016/S1470-2045(12)70412-6)
Supplement: Supplementary appendix [file mmc1.pdf]

THE LANCET Oncology

Supplementary appendix

This appendix formed part of the original submission and has been peer reviewed. We post it as supplied by the authors.

Supplement to: Lee SM, Khan I, Upadhyay S, et al. First-line erlotinib in patients with advanced non-small-cell lung cancer unsuitable for chemotherapy (TOPICAL): a double-blind, placebo-controlled, phase 3 trial. *Lancet Oncol* 2012; published online Oct 16. http://dx.doi.org/10.1016/S1470-2045(12)70412-6.

Participating clinicians and centres:

Addenbrookes Hospital (Hugo Ford); Aintree Hospitals NHS Trust (Chinnamani Eswar); Bangor (Ysbyty Gwynedd) (Nick Stuart); Beatson Oncology Centre Western Infirmary (David Dunlop); Blackpool Victoria Hospital (Andrew Hindley); Bradford Royal Infirmary (Andrew Conn); Bristol Haematology & Oncology Centre (Stephen Falk); ,Bronglais General Hospital (Alan Axford); Burnley General Hospital (Wiebke Appel); Castle Hill Hospital (Michael Lind); Charing Cross Hospital (Conrad Lewanski); Cheltenham General Hospital (David Farrugia); Clatterbridge Centre for Oncology (Ernie Marshall); Countess of Chester Hospital (Joe Maguire); Diana, Princess of Wales Hospital (Sunil Upadhyay); Doncaster Royal Infirmary (Matthew Hatton); Essex County Hospital (William Pratt); Falkirk & District Royal Infirmary (David Dunlop); Furness General Hospital (Geraldine Skailes); Guy's Hospital (Rohit Lal); Hairmyres Hospital (Mohammed Rizwanullah); Harrogate District Hospital (Samual Chen); Hereford County Hospital (Nick Reed); Huddersfield Royal Infirmary (Barbara Crosse); Inverclyde Royal Hospital (Richard Jones); Ipswich Hospital (J Morgan); James Cook University Hospital (Clive Peedell); Kent & Canterbury Hospital (Russell Burcombe); King's Mill Hospital (Karen Foweraker); Leicester Royal Infirmary (Gill Thomas); Maidstone Hospital (Henry Taylor); Monklands Hospital (NHS Lanarkshire) (Vivienne Maclaren); Mount Vernon (Jeanette Dickson); Norfolk and Norwich Hospital (WMC Martin); North Devon District Hospital (Mark Napier); North Middlesex Hospital (David Chao); North Tyneside Hospital (Jill Gardinerl); Nottingham City Hospital (Vanessa Potter); Poole Hospital (Dorset Cancer Centre) (Virginia Laurence); Queen Alexandra Hospital - Portsmouth (Tim Gulliford); Queen's Hospital, Burton (A D Chetiyawardana); Raigmore Hospital (Carol MacGregor); Royal Berkshire Hospital (Richard Brown); Royal Bournemouth Hospital (Tom Geldart); Royal Cornwall Hospital (Matthew Collinson); Royal Derby Hospital (previously Derbyshire Royal) (Dakshinamoorthy MuthuKumar); Royal Devon & Exeter Hospital (Elizabeth Toy); Royal Gwent Hospital (Alison Brewster); Royal Lancaster Infirmary (Geraldine Skailes); Royal Preston Hospital (Geraldine Skailes); Royal Surrey County Hospital (Gary Middleton); Scarborough Hospital (Amandeep Dhadda); Scunthorpe General Hospital (Sunil Upadhyay); Southampton General Hospital(Christian Ottensmeier); Southport & Formby District General Hospital (Pooja Jain); St Bartholomew's Hospital (Paula Wells); St George's Hospital, London (Tim Benepal); St Mary's Hospital - Isle of Wight (Christopher Baughan); St Mary's Hospital - London (C Lewanski); Stoke Mandeville Hospital (Nicholas Bates); Sunderland Royal Hospital (Andrew Hughes); Torbay Hospital (Elizabeth Toy); University College London Hospitals (Siow Ming Lee); University Hospital of North Durham (Rhona McMenemin); Walsgrave Hospital (Mark Hocking); Wansbeck General Hospital (Paula Mulvenna); Warrington Hospital (Ramani Vidhyasagar); Warwick Hospital (Caroline Humber); Weston Park Hospital (Penella Woll); Wexham Park Hospital (James Gildersleve); Whiston Hospital (David Marshall); Whittington Hospital (Siow Ming Lee); William Harvey Hospital (Russell Burcombe); Withybush,Hospital, (Vallipuram Vigneswaran); Worcestershire Royal Hospital (David Farrugia); Worthing Hospital (G Newman); Yeovil District Hospital (Stephen Falk); York Hospital (David Bottomley)

Supplementary figures and tables – TOPICAL trial

Supplementary Figure 1: Forest plots according to patients who did or did not develop first cycle rash in relation to pre-specified subgroups (baseline characteristics). All HRs are for erlotinib with or without rash vs. placebo

Overall survival

Patients with erlotinib rash

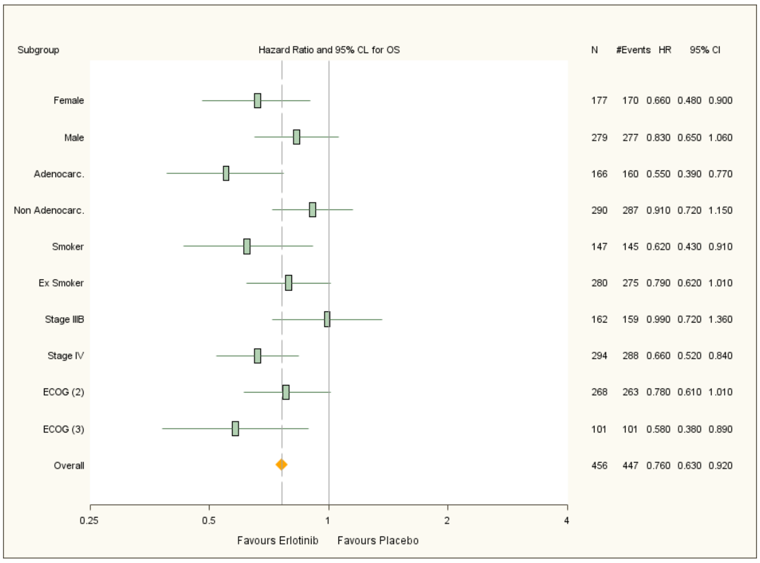

Patients without erlotinib rash

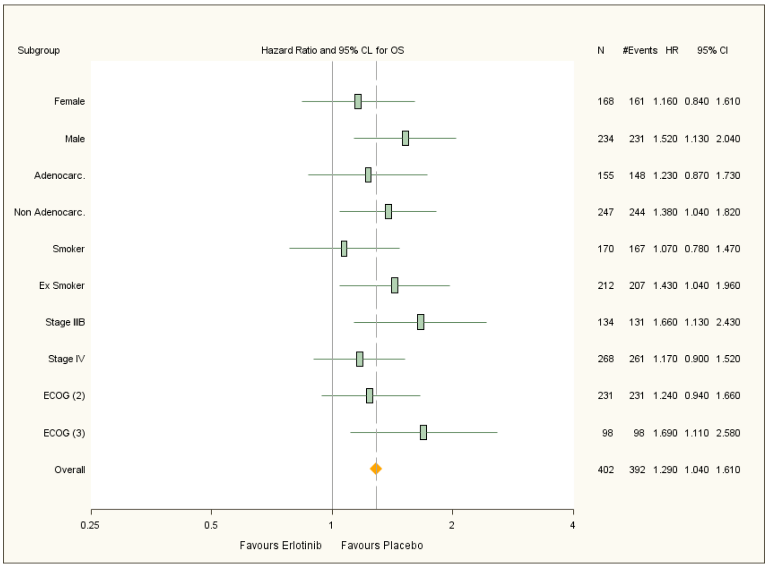

Progression-free survival

Patients with erlotinib rash

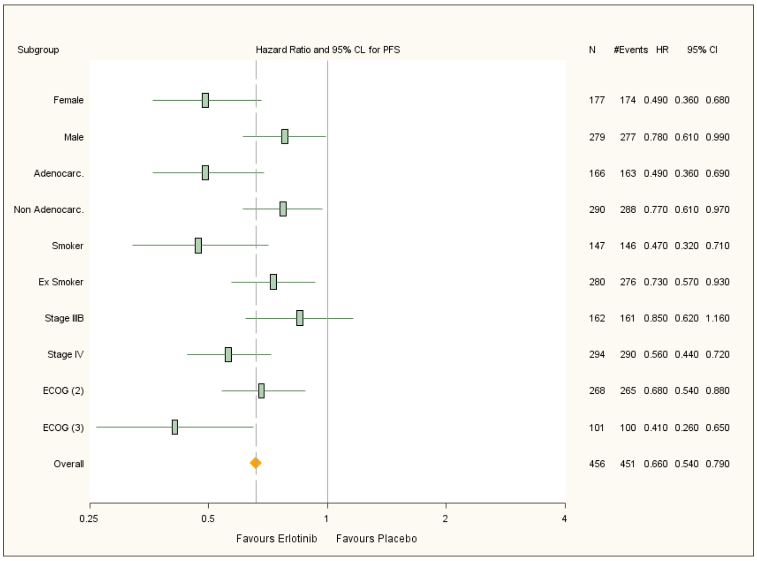

Patients without erlotinib rash

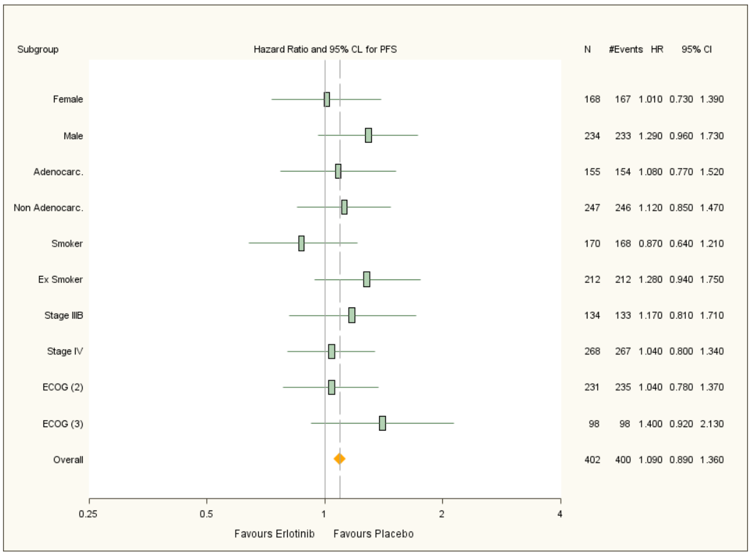

Supplementary Figure 2: Overall survival (OS) and progression-free survival (PFS) according to whether patients on erlotinib developed first-cycle rash or not (E refers to number of events) - excluding EGFR mutant-positive.

a) Overall Survival

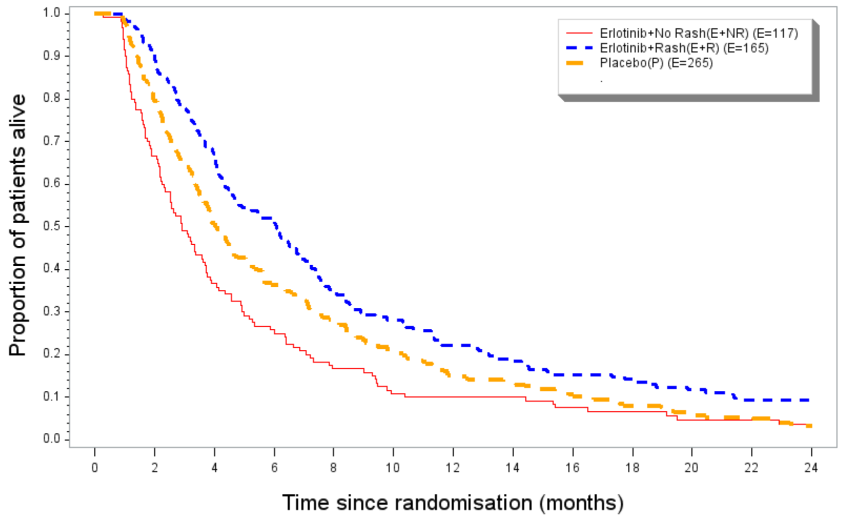

Patients at risk

E+NR	120	80	44	31	20	13	12	12	9	8	5	4	3
E+R	167	151	112	86	58	47	36	30	25	23	19	15	14
P	269	214	137	98	72	53	40	35	28	21	16	14	8

b) Progression Free Survival

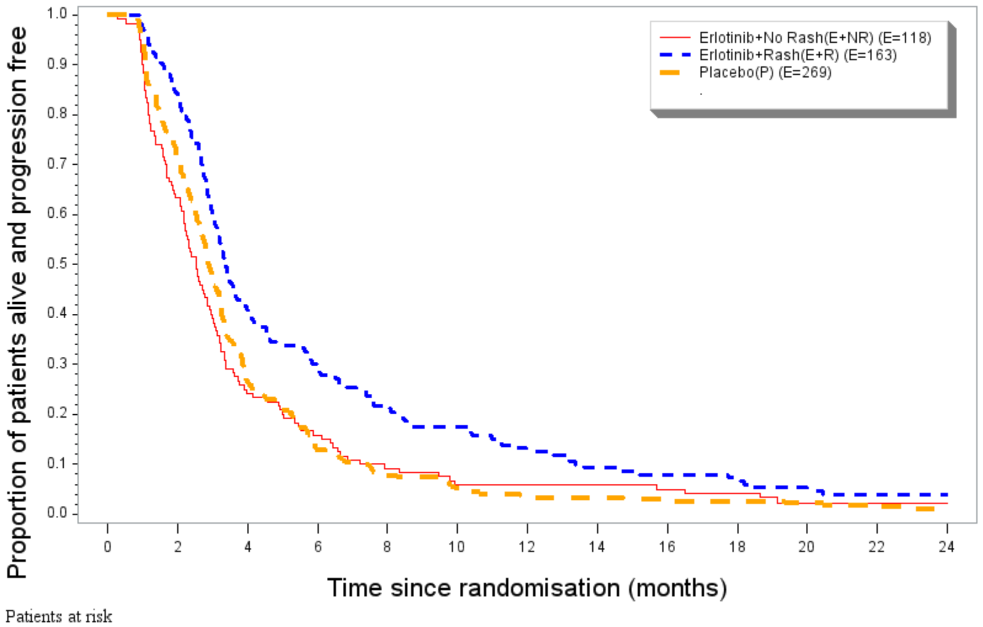

Patients at risk

E+NR	120	76	29	19	11	7	7	7	6	5	2	1	1
E+R	167	141	68	48	36	29	19	14	12	11	7	6	5
P	269	193	71	35	21	14	10	9	8	7	6	5	3

Supplementary Table 1. Compliance to study drug

**Compliance (%)**[1]	Erlotinib (N=350)	Placebo (N=320)
Median (%) (range)	88 (0-100)	86 (0-100)
$\geq$75% (n %)	204 (58)	203 (63)
<75% (n %)	124 (35)	105 (33)
unknown[2]	22 (6)	12 (4)

Compliance	Erlotinib rash	Erlotinib no rash	Placebo
≥75%	62%	56%	63%
<75%	34%	40%	33%

1: Calculated by the total number of tablets taken (equivalent to number of days when taking study drug) as recorded in the case report form (CRF) divided by the time from randomization to either death, progression or when treatment was stopped early, and expressed as a percentage.

2: Patients known to have started study drug but details of when it started or stopped were missing (17 Erlotinib and 10 Placebo).

Supplementary Table 2. Tumour Response

**Overall Response**	**Erlotinib (N=350)**	**Placebo (N=320)**
	n (%)	n (%)
Complete Response (CR)	3 (0.9)	0
Partial Response (PR)	12 (3.4)	7 (2.2)
Stable Disease (SD)	19 (5.4)	13 (4.1)
Progressive Disease (PD)	261 (74.6)	242 (75.6)
Not Evaluable	55 (15.7)	58 (18.1)

Supplementary Table 3. Quality of Life among all patients

**QLQC-30**	Erlotinib (N=350)	Placebo (N=320)	Difference (99% CI)
**Global Health Status**	50.87	50.18	0.69 (-2.05, 3.43)
**Functional Scales**			
Physical Functioning	53.90	50.79	**3.11 (0.48, 5.75)***
Role Functioning	49.09	46.77	2.32 (-1.80, 6.44)
Emotional Functioning	74.93	72.67	2.26 (-0.48, 5.00)
Cognitive Functioning	77.96	74.28	**3.68 (0.88, 6.49)***
Social Functioning	66.50	65.53	0.97 (-2.97, 4.92)
**Symptom Scales**			
Fatigue	50.38	49.92	0.46 (-2.87, 3.79)
Nausea / Vomiting	12.18	10.10	2.08 (-0.38, 4.54)
Pain	23.29	27.39	**-4.10 (-7.49, -0.71)***
Dyspnoea	48.18	55.15	**-6.97 (-10.7, -3.25)***
Insomnia	28.19	28.74	-0.55 (-4.32, 3.22)
Appetite loss	39.31	31.73	**7.58 (3.18, 11.99)***
Constipation	16.42	25.81	**-9.38 (-12.8, -5.97)***
Diarrhea	24.30	9.22	**15.08 (11.72,18.44)***
Financial Problems	7.89	11.79	**-3.90 (-6.40, -1.41)***
**Lung Specific symptoms (LC-14)**			
Sore Mouth	16.04	9.61	**6.43 (3.04, 9.82)***
Coughing	41.29	41.85	-0.56 (-3.73, 2.62)
Coughing up blood	5.77	3.83	1.94 (-0.13, 4.02)
Dyspnoea	41.01	44.38	**-3.37 (-6.58,-0.17)***
Dysphagia	9.26	9.33	-0.08 (-2.66, 2.50)
Hoarsness	16.86	20.52	**-3.66 (-7.16,-0.16)***
Perhiperal neuropathy	12.95	12.72	0.23 (-2.16, 2.63)
Pain in chest	12.86	18.72	**-5.86 (-8.87,-2.84)***
Hair Loss	17.26	4.66	**12.59 (9.83,15.36)**
Upset by hair loss	59.58	35.93	23.65 (-13.8,61.09)

*statistically significant at the 1% level

Scores range from 0 to 100 for QLQC-30 and LC-14 endpoints. For the global health and functional scales 0 indicates poor health and 100 good health. For all other scales, 0 indicates no symptoms and 100 high level of symptoms.

For the global health and functional scales a positive difference indicates improvement in QoL for erlotinib compared with placebo. For all other scales, a negative difference indicates improvements in QoL with erlotinib compared to placebo.

There was a treatment by time interaction for global health stats (p=0.01), role functioning (p=0.08), social functioning (P=0.04) and diarrhea (p=0.01).

Supplementary Table 4. Adverse events among patients who started erlotinib, and whether or not they developed first cycle rash

	Erlotinib+Rash (N=178)# n (%)	Erlotinib +No Rash (N=124)# n (%)	Placebo (N=278)# n (%)	P-value E+R[1]	P-value E+NR[2]
**Any Adverse event (maximum grade)**					
1	1 (0.6)	12 (10)	15 (5)		
2	9 (5)	15 (12)	30 (11)		
3	85 (48)	44 (36)	98 (35)		
4	83 (47)	37 (30)	119 (43)		
Any (grade 1-4)	178 (100)	108 (87)	262 (94)	0.002	0.12
Any (grade 3-4)	168 (94)	81 (65)	217 (78)	0.29	0.16
Any (grade 3-4) - excluding Rash & Diarrhea	88 (49)	73 (59)	213 (77)	<0.001	<0.01
**Rash (first cycle)**					
No rash / Grade 0 observed	0	124 (100)	263 (95)		
A (erythema alone)	54(30)	0	13 (5)	0.001	0.11
B (erythema with papules)	64(36)	0	2 (1)	<0.001	0.32
C (erythema with papules & pustules)	52(29)	0	0	<0.001	0.80
D (erythema with papules & confluent pustules)	8(5)	0	0	<0.001	0.13
**Dyspnoea**					
Grade 3 (dyspnoea on walking ≤100 yards)	59(33)	32(26)	87(31)		
Grade 4 (dyspnoea on mild exertion)	72(40)	33(27)	112(40)		
**Grade 3 – 4**	130(73)	66(53)	199(72)	0.58	0.004
**Specific adverse events (grade 3 or 4 only)**					
Fatigue	53(30)	24(19)	72(26)		
Diarrhea	20(11)	8(6)	4(1)	<0.001	0.006
Anorexia	14(8)	3(2)	15(5)		
Anaemia	4(2)	2(7)	3(1)		
Nausea	3(2)	2(7)	6(2)		
Pneumonitis	5(3)	0	1(0.4)		
Rigor chills	2(1)	2(2)	0		
Stomatitis	3(2)	1(2)	0		
Ocular	3(2)	0	0		
Constipation	1(0.6)	0	5(2)	0.26	0.13
Headache	0	0	2(0.7)		

1: E+Rash Vs Placebo 2: E+Non Rash vs Placebo
E+Rash: Erlotinib + Rash; E+Non Rash: Erlotinib and No Rash
Of the 334 Erlotinib patients known to have started study drug (Table 2), 32 had died before the assessment of 1st cycle could be made and are therefore excluded from the above table (leaving 302 patients, of which 178 had first cycle rash and 124 did not).Similarlyfor 35 patients among the 313 on Placebo (leaving 278 for the above analyses).

Supplementary Table 5. Distribution of the number of adverse events per patient; overall and according to rash / non rash (based on those who started study drug)

**Number of events**	**Erlotinib (E) (N=334)#**	**Placebo (N=313)#**	**E+Rash (N=178)##**	**E+No Rash (N=124)##**	**Placebo (N=278)##**
	n (%)	n (%)	n (%)	n (%)	n (%)
Any grade					
1	11 (3)	11 (4)	0	11 (9)	11 (4)
2	8 (2)	7 (2)	1 (0.6)	7 (6)	7 (3)
3	13 (4)	12 (4)	4 (2)	9 (7)	12 (4)
≥4	254 (76)	232 (74)	173 (97)	81 (65)	232 (83)
Grades 3-4					
1	54 (16)	52 (17)	27 (15)	27 (22)	52 (19)
2	39 (12)	40 (13)	23 (13)	16 (13)	40 (14)
3	38 (11)	26 (8)	23 (13)	15 (12)	26 (9)
≥4	118 (35)	99 (32)	95 (53)	23 (19)	99 (36)
Grades 3-4 (excluding 1st cycle rash)					
1	53 (16)	52 (17)	26 (15)	27 (22)	52 (19)
2	39 (12)	40 (13)	25 (14)	16 (13)	40 (14)
3	38 (12)	26 (8)	26 (15)	15 (12)	26 (9)
≥4	107 (32)	99 (32)	84 (47)	23 (19)	99 (36)

#Patients known to have started study drug (see Table 2)
Taken from Supplementary Table 4

Supplementary Table 6. Quality of Life among all patients, and according to whether or not they developed first cycle rash. The table shows the mean difference in QoL scores between erlotinib (E) and placebo

**QLQC-30**	Overall	E+Rash vs Placebo	E+No Rash Vs Placebo
	Difference (99% CI)	Difference (99% CI)	Difference (99% CI)
**Global Health Status**	0.69 (-2.05, 3.43)	4.23 (-1.19, 9.64)	-2.53 (-9.52, 4.47)
**Functional Scales**			
Physical Functioning	**3.11 (0.48, 5.75)***	1.29 (-6.28,8.86)	-7.47 (-17.40, 2.45)
Role Functioning	2.32 (-1.80, 6.44)	2.10 (-3.21,7.40)	-0.81 (-7.67, 6.06)
Emotional Functioning	2.26 (-0.48, 5.00)	2.91 (-2.59,8.41)	2.16 (-5.00, 9.31)
Cognitive Functioning	**3.68 (0.88, 6.49)***	0.10 (-7.69, 7.89)	-3.18 (-13.18, 6.83)
Social Functioning	0.97 (-2.97, 4.92)	0.63 (-4.41,5.67)	-2.47 (-9.05, 4.11)
**Symptom Scales**			
Fatigue	0.46 (-2.87, 3.79)	-0.67 (-7.10,5.76)	4.93 (-3.40, 13.26)
Nausea / Vomiting	2.08 (-0.38, 4.54)	3.26 (-1.54,8.05)	1.35 (-4.91, 7.62)
Pain	**-4.10 (-7.49, -0.71)***	-1.73 (-8.05,4.59)	-0.66 (-8.88, 7.55)
Dyspnoea	**-6.97 (-10.7, -3.25)***	**-9.29 (-16.21,-2.37)***	4.04 (-5.05,13.12)
Insomnia	-0.55 (-4.32, 3.22)	-3.48 (-10.58,3.63)	**13.17 (3.92,22.42)***
Appetite loss	**7.58 (3.18, 11.99)***	7.07 (-1.35,15.48)	8.02 (-2.95,18.99)
Constipation	**-9.38 (-12.8, -5.97)***	**-10.73 (-17.16,-4.30)***	**-10.27 (-18.68,-1.85)***
Diarrhea	**15.08 (11.72,18.44)***	**17.79 (11.75,23.83)***	**16.23 (8.30,24.15)***
Financial Problems	**-3.90 (-6.40, -1.41)***	-4.53 (-9.48,0.42)	2.67 (-3.72, 9.06)
**Lung Specific symptoms (LC-14)**			
Sore Mouth	**6.43 (3.04, 9.82)***	**8.18 (1.66, 14.70)***	5.30 (-3.26,13.86)
Coughing	-0.56 (-3.73, 2.62)	-2.69 (-9.10, 3.72)	0.51 (-7.86,8.88)
Coughing up blood	1.94 (-0.13, 4.02)	2.50 (-2.07, 7.07)	1.76 (-4.26, 7.78)
Dyspnoea	**-3.37 (-6.58,-0.17)***	-5.98 (-12.76, 0.81)	0.57 (-8.39, 9.54)
Dysphagia	-0.08 (-2.66, 2.50)	0.69 (-4.51, 5.88)	1.35 (-5.49, 8.18)
Hoarsness	**-3.66 (-7.16,-0.16)***	-4.11 (-11.31, 3.08)	-2.77 (-12.10, 6.56)
Perhiperal neuropathy	0.23 (-2.16, 2.63)	-0.53 (-6.30, 5.25)	4.61 (-2.93, 12.15)
Pain in chest	**-5.86 (-8.87,-2.84)***	**-6.72 (-12.68,-0.77)***	-0.86 (-8.68,6.97)
Hair Loss	**12.59 (9.83,15.36)**	**10.60 (5.15, 16.05)***	6.01 (-1.19,13.21)
Upset by hair loss	23.65 (-13.8,61.09)	26.23 (-34.74, 87.21)	21.48 (-47.68,90.65)

*statistically significant at the 1% level

Scores range from 0 to 100 for QLQC-30 and LC-14 endpoints. For the global health and functional scales 0 indicates poor health and 100 good health. For all other scales, 0 indicates no symptoms and 100 high level of symptoms.

For the global health and functional scales a positive difference indicates improvement in QoL for erlotinib compared with placebo. For all other scales, a negative difference indicates improvements in QoL with erlotinib compared to placebo.

There was a treatment by time interaction for global health stats (p=0.01), role functioning (p=0.08), social functioning (P=0.04) and diarrhea (p=0.01).

Supplementary Table 7. Hazard ratios (compared to placebo) for OS and PFS according to histology and gender

	Erlotinib+Rash			Erlotinib +No Rash		
	HR	95%CI	P-value	HR	95%CI	P-value
**Adenocarcinoma**						
OS						
Females	0.55	0.34-0.88	0.014	1.12	0.71-1.78	0.605
Males	0.53	0.33-0.86	0.010	1.61	0.94-2.76	0.08
PFS						
Females	0.47	0.29-0.76	0.0019	1.06	0.68-1.67	0.78
Males	0.52	0.32-0.84	0.007	1.61	0.94-2.76	0.08
**Non-adenocarcinoma**						
OS						
Females	0.76	0.49-1.15	0.19	1.36	0.85-2.17	0.20
Males	0.82	0.72-1.29	0.82	1.48	1.04-2.10	0.027
PFS						
Females	0.55	0.36-0.85	0.007	0.94	0.59-1.49	0.79
Males	0.88	0.66-1.18	0.41	1.30	0.92-1.84	0.13

*all HRs compared to placebo
